# Protective immunity against *Trichinella spiralis* infection induced by TsNd vaccine in mice

**DOI:** 10.1186/s13071-015-0791-8

**Published:** 2015-03-28

**Authors:** Pei Liu, Jing Cui, Ruo Dan Liu, Min Wang, Peng Jiang, Li Na Liu, Shao Rong Long, Ling Ge Li, Shuai Bing Zhang, Xin Zhuo Zhang, Zhong Quan Wang

**Affiliations:** Department of Parasitology, Medical College, Zhengzhou University, 40 Daxue Road, Zhengzhou, 450052 P. R. China; Department of Infection Control, The Second People’s Hospital of Zhengzhou City, Zhengzhou, 450000 P. R. China

**Keywords:** *Trichinella spiralis*, Nudix hydrolase (Nd), DNA vaccine, Th1/Th2

## Abstract

**Background:**

We have previously reported that *Trichinella spiralis* Nudix hydrolase (TsNd) bound to intestinal epithelial cells (IECs), and vaccination of mice with recombinant TsNd protein (rTsNd) produced a partial protective immunity. The aim of this study was to investigate the immune protection induced by TsNd DNA vaccine.

**Methods:**

The full-length cDNA sequence of TsNd gene was cloned into pcDNA3.1 and used to immunize BALB/c mice by intramuscular injection. Transcription and expression of TsNd were detected by RT-PCR and IFT. The levels of specific IgA, IgG, IgG1 and IgG2a, and cytokines were assayed by ELISA at weeks 0, 6 and 8 post-immunization. The immune protection of TsNd DNA vaccine against challenge infection was investigated.

**Results:**

Immunization of mice with TsNd DNA elicited a systemic Th1/Th2 immune response and a local mucosal IgA response. The *in vitro* transcription and expression of TsNd gene was observed at all developmental stages of *T. spiralis* (ML, IIL, AW and NBL). Anti-rTsNd IgG levels were increased after immunization and levels of IgG1 were obviously higher than that of IgG2a. Intestinal specific IgA levels of immunized mice were significantly higher than those of vector and PBS control mice. Cytokine profiling also showed a significant increase in Th1 (IFN-γ, IL-2) and Th2 (IL-4, 10) responses in splenocytes of immunized mice on stimulation with rTsNd. Vaccination of mice with pcDNA3.1-TsNd displayed a 40.44% reduction in adult worms and a 53.9% reduction in larval burden.

**Conclusions:**

TsNd DNA induced a mixed Th1/Th2 immune response and partial protection against *T. spiralis* infection in mice.

## Background

*Trichinella spiralis* is a parasitic nematode that infects humans and other mammals [1]. Trichinellosis, caused by the consumption of raw or undercooked meat contaminated with *Trichinella* infective muscle larvae, remains an important food-borne disease with a nearly worldwide distribution [2]. In the past several decades, many outbreaks of human trichinellosis have been reported in different areas of the world [3]. From 2004 to 2009, 15 outbreaks of human trichinellosis, with 1387 cases and 4 deaths, were reported in China [4]. The occurrence of trichinellosis in humans is strictly related to cultural food practices, pork is the most important source of human *Trichinella* infection in China [5]. Trichinellosis is not only a public health hazard but also an economic problem in porcine animal production and food safety [6]. Due to the predominantly zoonotic importance of *Trichinella* infection, the development of vaccines capable of preventing swine from becoming infected is a promising measure for control of trichinellosis [7-9].

*T. spiralis* muscle larvae (ML) are released from the muscle tissue in the stomach by the digestive enzymes, and activated into the intestine infective larvae (IIL) by exposure to intestinal contents or bile. Then the IIL invade host’s intestinal epithelium where the larvae develop into adult worms (AW) that mate and reproduce the next generation of larvae [10]. Therefore, the intestinal mucosa is the first natural barrier in protecting the host against *Trichinella* infection. In our previous study, *T. spiralis* Nudix hydrolase (TsNd) protein binding to normal mouse intestinal epithelial cells (IECs) were identified by screening a T7 phage display cDNA library from *T. spiralis* IIL [11]. TsNd gene (GenBank accession No. EU263318.1) was also an up-regulated gene in IIL compared to ML, which was identified by using suppression subtractive hybridization (SSH) and confirmed by real-time PCR [12]. The TsNd gene was about 1248 bp. The predicted open reading frame (ORF) of TsNd encodes 415 amino acids with a molecular weight of 46 kDa and an isoelectric point (pI) of 8.85. Conserved domain analysis of TsNd revealed there was one Nudix motif located at 226–244 aa. The vaccination of mice with recombinant TsNd protein (rTsNd) produced a partial protective immunity against *T. spiralis* infection [13].

In recent years, many promising results and significant protection have been reported for viral, bacterial, and parasitic infections using the DNA heterologous prime-boost vaccination [14-16]. In this study, to observe the immune protection induced by TsNd DNA vaccine, the full-length cDNA sequence of TsNd gene was cloned into the eukaryotic expression vector pcDNA3.1 and used to immunize BALB/c mice by intramuscular injection. In addition, the humoral and cellular immune responses elicited by the DNA vaccine were investigated.

## Methods

### Ethics statement

This study was carried out in strict accordance with the National Guidelines for Experimental Animal Welfare (MOST of People’s Republic of China, 2006). All animal procedures reported herein were reviewed and approved by the Zhengzhou University Animal Care and Use Committee (Permission No. SYXK 2012–0009).

### Parasite and experimental animals

The isolate (ISS534) of *T. spiralis* used in this study was obtained from domestic pigs in Nanyang, Henan Province, China. The *Trichinella* isolate was maintained by serial passage in Kunming mice every 6–8 months. Specific pathogen-free (SPF) female BALB/c mice aged 6–8 weeks were obtained from the Laboratory Animal Center of the Experimental Animal Center of Henan Province.

### Collection of worms

*T. spiralis* ML from infected mice at 35 days post-infection (dpi) were recovered by digestion of carcasses with 0.33% pepsin (1:31000; Sigma) and 1% HCl [17]. The IIL was collected from the mouse small intestines at 6 hours post-infection (hpi). Adult worms (AW) were isolated from the small intestines of infected mice at 7 dpi [18]. The newborn larvae (NBL) were collected from female adult worms cultured in RPMI-1640 medium containing 10% fetal bovine serum (FBS; Gibco) in 5% CO_2_ at 37°C for 24 h [19].

### Plasmid construction and sequence analysis of recombinant expression plasmids

The full-length TsNd gene (GenBank accession No. EU263318.1) was obtained by PCR amplification using the following primers: 5-A***GGATCC***GCCACCATGTTTTACTTGGTAA CG-3 (forward), 5-A***CTCGAG***TTACCAAGTGTGTTGCAAAGCAATC-3 (reverse). The BamHI and XhoI sites are bold and italicized. DNA encoding the full-length TsNd was cloned into the eukaryotic expression vector pcDNA3.1 as DNA vaccine (pcDNA3.1-TsNd). An artificial Kozak sequence (GCCACC) was included in sense primers to permit efficient initiation of translation [20]. Amplicons were ligated into the expression vector pcDNA3.1 (Invitrogen, USA) and transformed into *E. coli* DH5a (Invitrogen). Clones containing inserts of the expected size were selected by restriction digestion and subjected to DNA sequencing using T7 and BGH primers by Invitrogen (Invitrogen Co. Ltd, Shanghai, China). DNA and predicted amino acid sequences were analyzed using Lasergene 7.1 software (DNASTAR Inc., Madison, Wisconsin, USA). The plasmid DNA was extracted by alkaline cleavage method [16].

### Expression and purification of recombinant TsNd protein (rTsNd)

The full-length TsNd gene was cloned into the expression vector pMAL-c2X (New England Biolabs, USA). The rTsNd protein was expressed in *E. coli* under 0.5 mM IPTG induction as described previously [13]. The rTsNd protein was purified by Amylose Pre-packed Column (NEB Ltd, China). The mouse anti-rTsNd serum was collected from the mice immunized with rTsNd obtained at one week after the last immunization as described previously [21].

### RT-PCR and immunofluorescent test (IFT)

The baby hamster kidney cells (BHK-21) were plated in 6-well tissue culture plates (Nunc) at 2 × 10^6^ cells per well in RPMI-1640 containing 5% FBS [22]. The recombinant pcDNA3.1-TsNd and pcDNA3.1 (1 × 10^8^/mL) were transfected into the BHK-21 cells with Lipofectamine 2000 (Invitrogen, USA) following the manufacturer’s instructions. For RT-PCR assays, total RNA was extracted from the transfected BHK-21 cells using TRIzol reagent (Invitrogen, USA) following the manufacturer’s instructions. The cDNA samples were obtained using a cDNA First Strand Synthesis Kit (Bioer Technology, China). Transcription of the TsNd gene in the transfected BHK-21 cells was analyzed by RT-PCR using specific primers listed above. For IFT, BHK-21 cells expressing TsNd proteins were cultured as monolayers and washed with PBS and fixed with 4% acetone at room temperature for 20 min. Then, cells were washed with PBS and permeabilized with 1% TritonX- 100 at 4°C for 10 min, after which the cells were further incubated with mouse anti-rTsNd serum (1:10 dilution) at 37°C for 1 h. After washing three times in PBS, the cells were incubated with a 1:50 dilution of FITC-labeled goat anti-mouse IgG (Santa Cruz, USA) in PBS at room temperature for 1 h, after washing three times in PBS, and examined under a fluorescent microscope (Olympus, Japan).

### Immunization schedule and sample collection

BALB/c mice were randomly divided into three groups of 40 animals each. Pre-immune sera were collected by tail bleeding 2 days prior to the first vaccination. The vaccine group of mice was intramuscularly injected with TsNd-pcDNA3.1. The control group was injected with pcDNA3.1 or PBS. The vaccination was administered four times at 2-week intervals. For intramuscular vaccination, mice were injected with 50 μg of pure plasmid DNA (in 50 μl of PBS) in the left and right quadriceps of legs [15]. At weeks 0, 6 and 8 post-immunization, ten mice from each group were euthanized; the serum, intestinal lavage fluid, and spleen were collected to evaluate the humoral and cellular immune responses.

### Evaluation of humoral immune responses by ELISA

The levels of the specific total IgG, IgG1 and IgG2a antibodies to TsNd in serum samples of immunized mice were determined by ELISA with rTsNd as described previously [23,24]. Briefly, microtiter plates (Nunc) were coated with rTsNd (2.5 μg/ml) in coating buffer overnight at 4°C, and blocked with 200 μl of PBS-0.1% Tween 20 (PBST) containing 5% skimmed milk. Then, 100 μl of serum samples with 1:100 dilution in PBS were added to each well and incubated at 37°C for 1 h. HRP-conjugated anti-mouse IgG, IgG1 or IgG2a (1:5000; Southern Biotechnology, USA) were added and incubated at 37°C for 1 h. The reactions were detected by addition of the substrate o-phenylenediamine dihydrochloride (OPD; Sigma) plus H_2_O_2_ and stopped with 50 μl/well of 2 M H_2_SO_4_. Absorbance at 490 nm was measured with a microplate reader (TECAN, Austria). All samples were run in duplicate.

### Recognition of the native TsNd in *T. spiralis* different stages by IFT

The recognition of the native TsNd in *T. spiralis* different developmental stages (ML, IIL, AW, and NBL) was observed by IFT as described previously [25,26]. The intact whole worms were fixed in aceton and further incubated with anti-rTsNd serum from mice immunized by TsNd DNA (1:10 dilution) at 37°C for 1 h. After being washed three times in PBS, the whole parasites were incubated with a 1:50 dilution of FITC-labeled goat anti-mouse IgG (Santa Cruz, USA), washed five times in PBS, and examined under a fluorescent microscope (Olympus, Japan).

### Measurement of total IgA and specific IgA in intestinal washings

To determine the specific IgA, the interior of the small intestine from immunized mice was washed twice with a total of 1 ml of cold PBS. After centrifugation at 800 g for 10 min, the supernatants of the intestinal washes were harvested. The specific anti-TsNd IgA was measured by standard ELISA using an rTsNd-coated plate. Total IgA was quantified by a sandwich ELISA using rabbit anti-mouse IgA antibody (Abcam, UK) as the capture antibody and HRP-conjugated goat anti-mouse IgA antibodies as the detection antibody. To compensate for variations in the efficiency of the recovery of secretory IgA antibodies among animals, the antigen-specific IgA in each sample was normalized to the total IgA present in the lavage.

### Cytokine assays

To examine the specific cellular immune responses, spleen cells were aseptically removed from immunized and control mice at weeks 0, 6 and 8 post-immunization, respectively [27]. The density of spleen cells obtained from each mouse was adjusted to 2 × 10^6^ cells/ml in complete RPMI-1640 containing 10% fetal bovine serum (FBS), 100U/ml penicillin and 100U/ml streptomycin. Spleen cells in 96-well plates were stimulated with the rTsNd (2 × 10^10^ pfu/ml) at 37°C for 48 h in a humidified 5% CO_2_ atmosphere. Supernatants were collected and cytokines (IL-2, IFN-γ, IL-4, and IL-10) were measured by ELISA [28]. Simultaneously, the cells were incubated with ConA as a positive control and RPMI 1640 medium alone as a negative control. Cytokine concentrations were determined by comparison with standard curves constructed with known amounts of the respective mouse recombinant cytokines. The Results were expressed in picograms per milliliter (pg/ml).

### Challenge infection

One week after the final vaccination, the remaining 60 mice from the 3 groups (20 mice for each group) were orally challenged with 300 *T. spiralis* ML. Ten mice from each group were euthanized 7 days after challenge and the numbers of intestinal adult worms were counted [23]. The muscle larvae were examined by artificial digestion from another 10 mice from each group 35 days after challenge [21]. The protective immunity was calculated as the worm reduction rate of recovered adults and larvae per gram (LPG) muscle from the immunized groups versus those from the control group [15,29].

### Statistical analysis

Data were expressed as the mean ± standard deviation. Intra-and intergroup statistical analyses were performed with one-way ANOVA (LSD test) using SPSS version 17.0 software. *P* < 0.05 was considered as statistically significant.

## Results

### Construction of recombinant pcDNA3.1-TsNd

**Figure 1:**
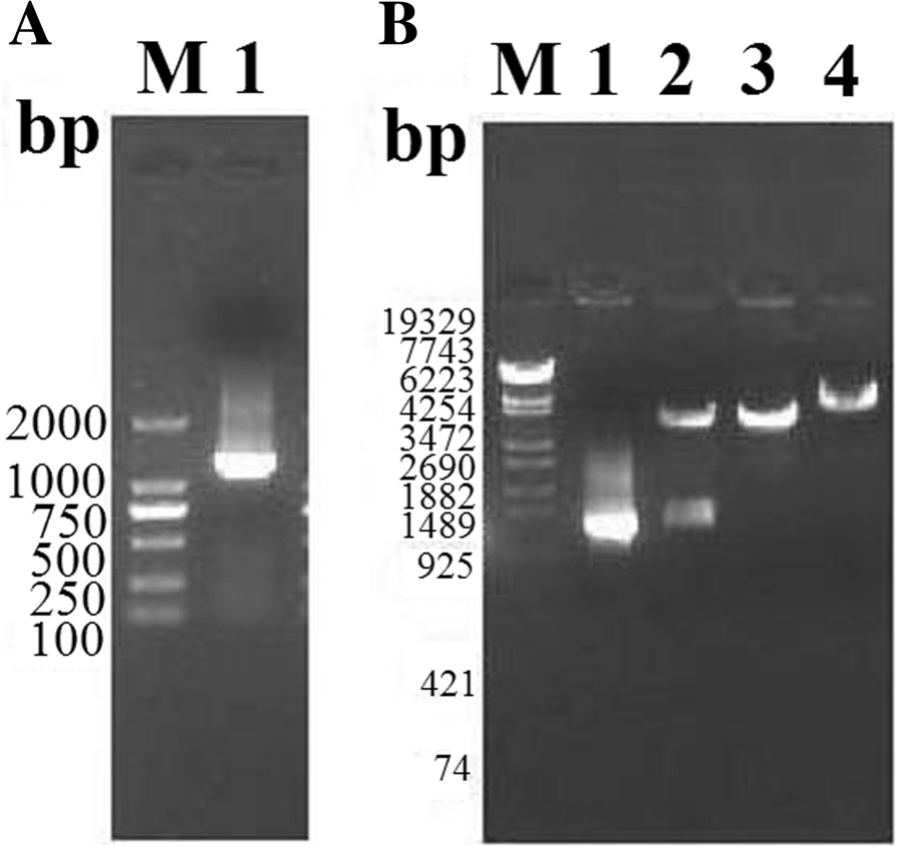
**PCR identification of TsNd gene and recombinant plasmid pcDNA3.1-TsNd digested. (A)** PCR identification of TsNd gene. M: DL2000 marker; 1: PCR product of pcDNA3.1-TsNd; **(B)** Restriction endonuclease digestion of recombinant plasmid pcDNA3.1-TsNd. M: DL2000 marker; lane 1: pcDNA3.1-TsNd; lane 2: pcDNA3.1-TsNd digested with BamHI/XhoI; lane 3: pcDNA3.1-TsNd digested with BamHI; lane 4: pcDNA3.1-TsNd digested with XhoI.

The full-length cDNA encoding TsNd was cloned into pcDNA3.1, the recombinant plasmid pcDNA3.1-TsNd was digested with BamHI/XhoI and the results revealed that the recombinant plasmid contained an insert of about 1248 bp (Figure 1). Sequence analysis indicated that the amplified fragment of TsNd consisted of 1248 bp, and the predicted ORF encoded a protein of 415 amino acid residues with a molecular mass of 46 kDa and 99.7% identity to the published sequence of TsNd in GenBank (EU263318.1).

### The *in vitro* transcription and expression of TsNd gene

**Figure 2:**
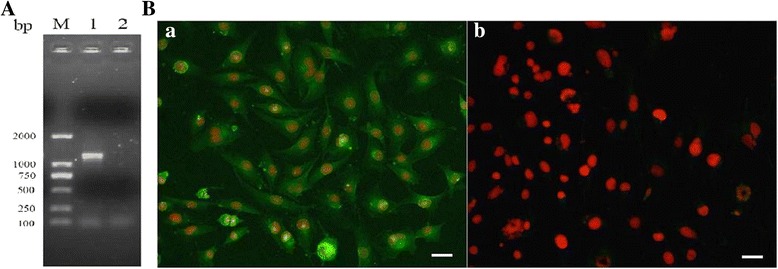
**Transcription and expression of recombinant pcDNA3.1-TsNd in BHK-21 cells. (A)** The *in vitro* transcription of TsNd gene in the BHK-21 cells was analyzed by RT-PCR. PCR products were analyzed on a 1% agarose gel with ethidiumbromide staining. M: DL2000 marker; 1: pcDNA3.1-TsNd group; 2: empty plasmid pcDNA3.1 group. **(B)** Expression of TsNd gene in the BHK-21 cells was detected by IFT using anti-rTsNd serum. **(a)** BHK-21 cells transfected with pcDNA3.1-TsNd, **(b)** BHK-21 cells transfected with pcDNA3.1. The scale bar is 10 μm.

The *in vitro* transcription of TsNd gene in the BHK-21 cells was analyzed by RT-PCR. PCR products were analyzed on a 1% agarose gel with ethidiumbromide staining. The amplified TsNd fragment was observed in pcDNA3.1-TsNd group, but the amplified fragment was not observed in pcDNA3.1 control group (Figure 2A). Expression of TsNd gene in BHK-21 cells was detected by IFT. The intense green fluorescent staining using anti-rTsNd serum was observed in BHK-21 cells transfected with pcDNA3.1-TsNd, but not detected in plasmid pcDNA3.1 control group (Figure 2B).

### Humoral immune responses induced by immunization with pcDNA3.1-TsNd

**Figure 3:**
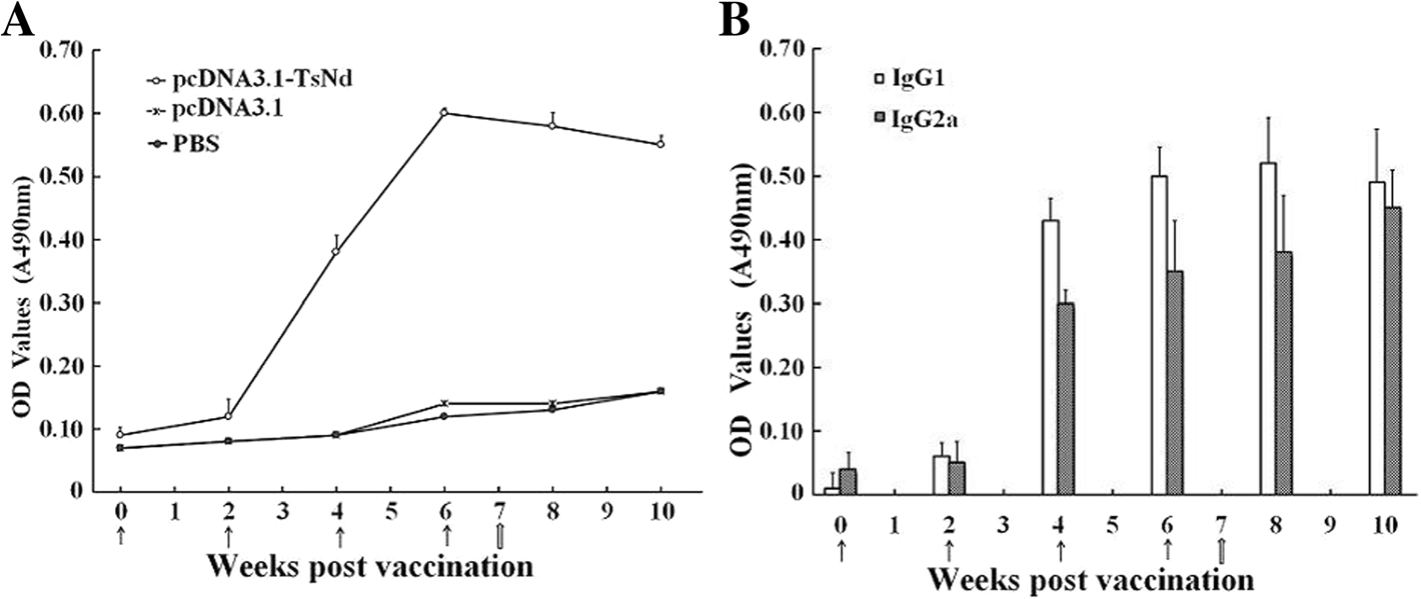
**Mouse IgG (A) and IgG subclass (B) responses to the immunization with pcDNA3.1-TsNd as measured by ELISA with rTsNd.** The OD values shown for each group are the mean ± standard deviation (SD) of antibody levels (n = 10). The immunization time points are marked as solid arrows (↑), and the challenge time is marked as empty arrow ().

The mouse sera collected at different time points after immunization were used to measure levels of specific anti-TsNd IgG and its subtype (IgG1 and IgG2a) antibodies. Anti-TsNd IgG levels in mice immunized with pcDNA3.1-TsNd were significantly increased following the second and third immunization. However, none of the mice vaccinated with pcDNA3.1 or PBS showed significantly detectable TsNd-specific IgG antibody responses (Figure 3A). In the immunized group with pcDNA3.1-TsNd (Figure 3B), the levels of IgG1 on week 4 and 6 after the first immunization were more obviously higher than that of IgG2a (t_4w_ = 24.756, t_6w_ = 30.163, *P* < 0.01). However, it was notable that IgG2a was induced on week 4 and 6. This suggests that vaccination with pcDNA3.1-TsNd elicited a Th1/Th2-mixed type of immune response with Th2 predominance.

### Recognition of the native TsNd at *T. spiralis* different stages by IFT

**Figure 4:**
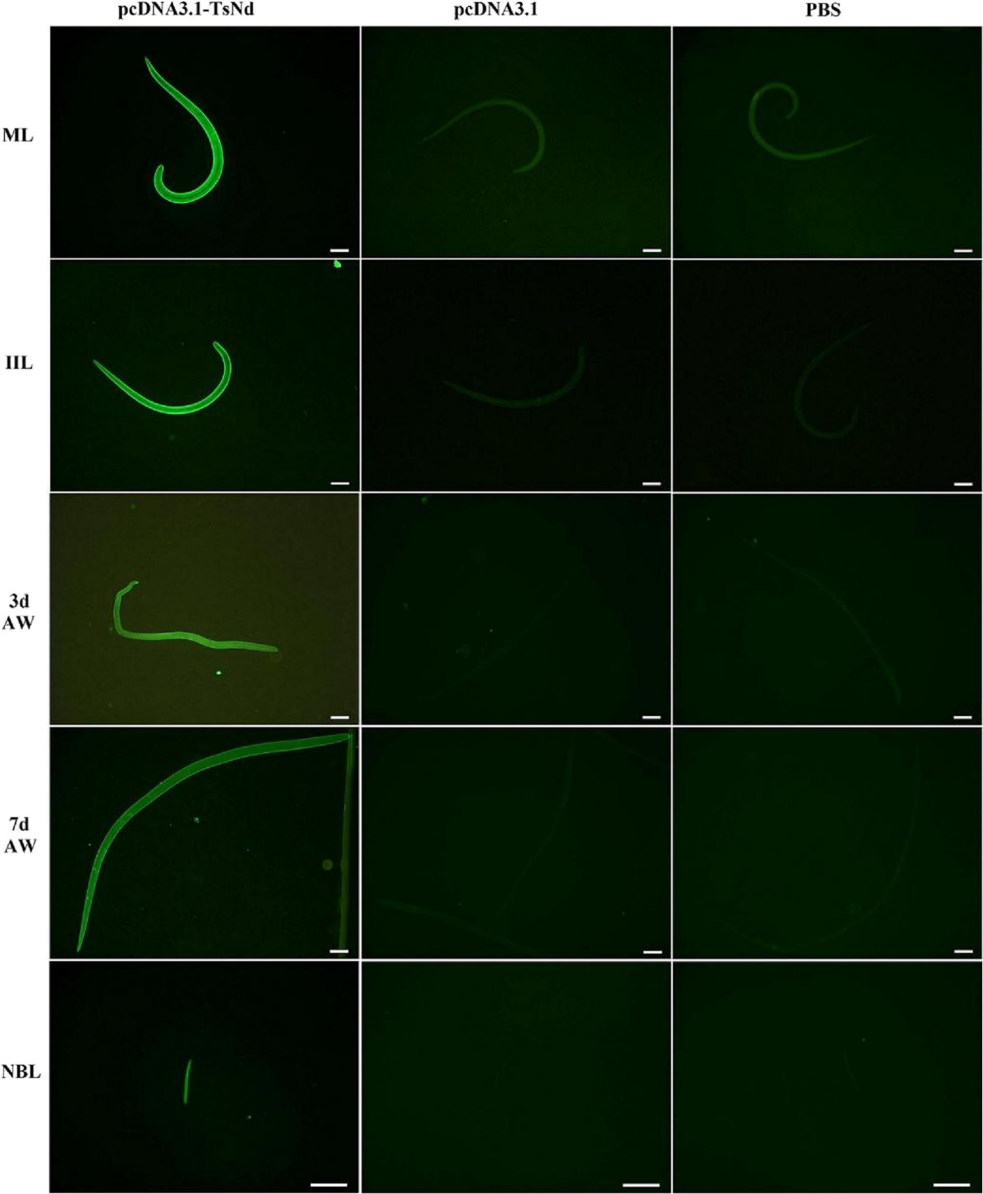
**Recognition of** ***T. spiralis*** **different developmental stages by IFT with serum IgG from mice vaccinated with pcDNA3.1-TsNd, empty plasmid pcDNA3.1and PBS.** Scale bar =10 μm.

The results of IFT with the whole parasites showed that the intense fluorescent staining using anti-rTsNd serum was found on the surface of all different developmental stages of *T. spiralis* (e.g., ML, IIL at 6 hpi, AW at 3 dpi, AW at 7 dpi and NBL). Serum from mice vaccinated with empty plasmid pcDNA3.1 or PBS did not recognize the *T. spiralis* different stages (Figure 4).

### Mucosal immune response

**Figure 5:**
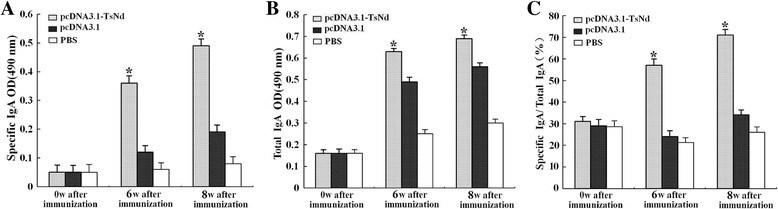
**Anti-TsNd-specific IgA (A), total IgA (B) and specific IgA/total IgA (C) in intestinal washings of mice immunized with pcDNA3.1-TsNd, vector or PBS.** Results are the mean ± standard deviation (SD) for 10 mice per group. Asterisks (*) indicate statistically significant differences (*P* < 0.05) compared to the empty plasmid pcDNA3.1 or PBS control group.

Intestinal total IgA levels were significantly increased in mice immunized with pcDNA3.1-TsNd compared with those immunized with vector alone or PBS (*P* < 0.05). Intestinal specific anti-TsNd IgA levels of mice immunized with pcDNA3.1-TsNd were obviously higher than those of immunized with vector alone or PBS (*P* <0.05). And statistical difference was also observed in ratio of specific IgA/total IgA in immunized group compared with those vaccinated with empty plasmid pcDNA3.1 alone or PBS (*P* <0.05) (Figure 5).

### Cytokine responses to the immunization with pcDNA3.1-TsNd

**Figure 6:**
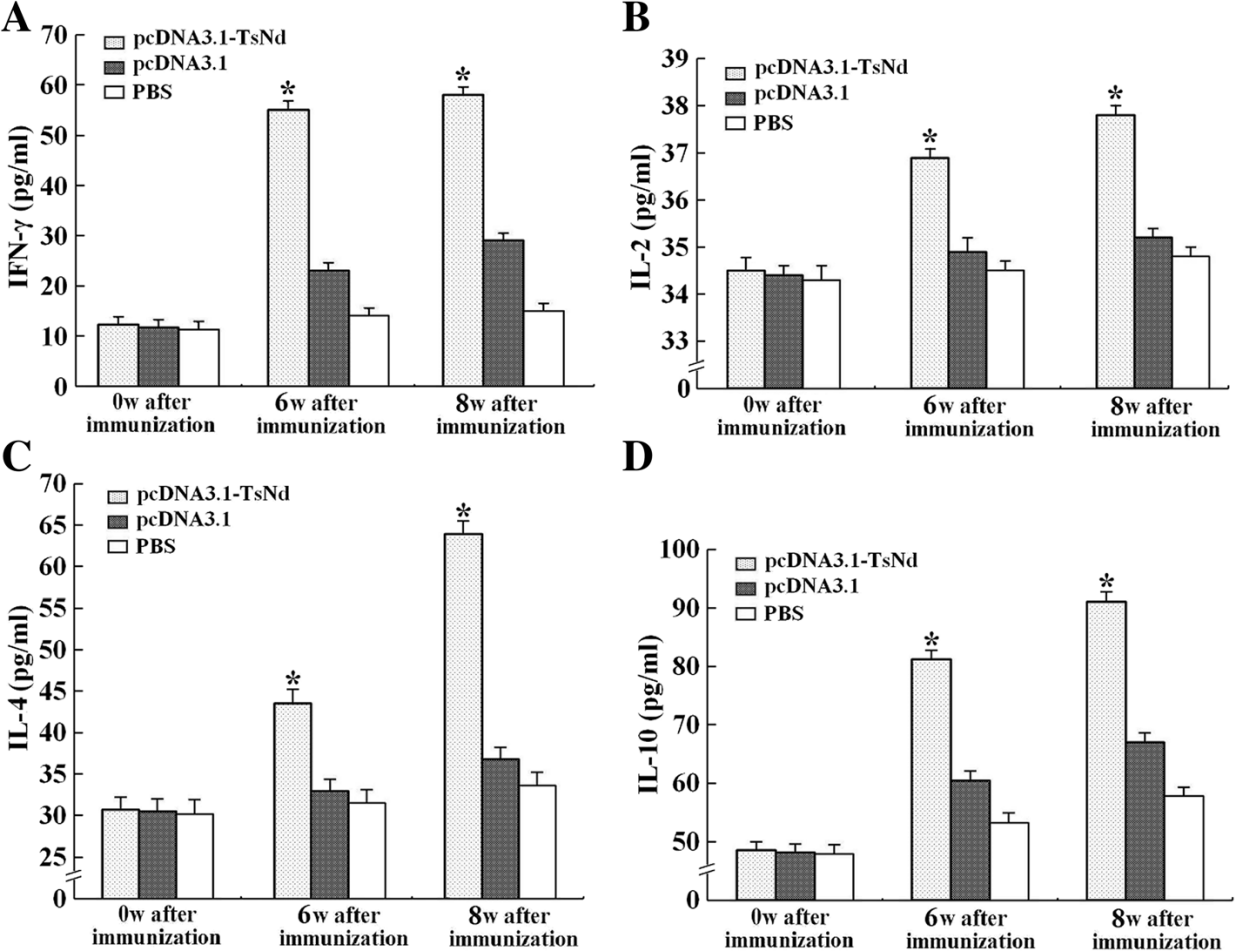
**Splenocyte-secreted IFN-γ (A) IL-2 (B), IL-4 (C)**, **and IL-10 (D) upon rTsNd stimulation were detected by ELISA.** Concentrations of cytokines detected in splenocyte supernatants after stimulation with the rTsNd for 48 h are shown. Data are presented as the mean cytokine concentrations ± standard deviation (SD) of 10 mice per group. *P < 0.05: indicate statistically significant differences with the empty plasmid pcDNA3.1 or PBS control group.

At week 6 after immunization, the spleen cells from mice immunized with pcDNA3.1-TsNd produced significantly higher levels of IFN-γ, IL-2, IL-4, and IL-10, compared with those before immunization (*P* < 0.01) (Figure 6). Moreover, the levels of four cytokines in immunized mice continued to increase at week 8 after immunization (one week after challenge). Compared with the empty plasmid pcDNA3.1 and PBS control groups, the levels of four cytokines in immunized mice were statistically obviously increased at week 6 and 8 after immunization (*P* <0.01), suggesting that the vaccination of mice with pcDNA3.1-TsNd induced the Th1/Th2-mixed type of immune response.

### Immune protection

**Figure 7:**
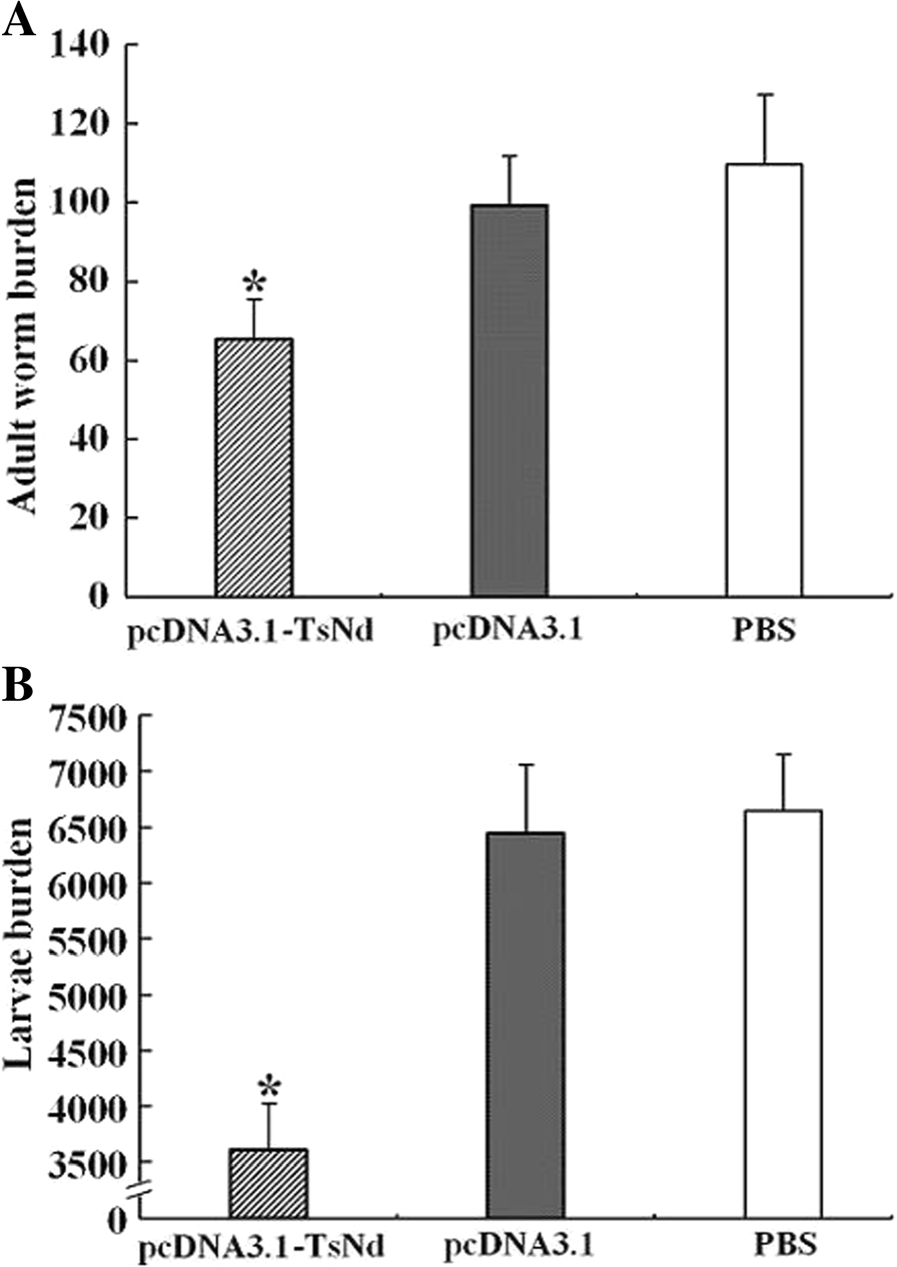
**The number of intestinal adult worms (A) and larvae per gram (LPG) of muscles (B) recovered from vaccinated mice after a challenge infection with 300** ***T. spiralis*** **larvae.** Results are presented as the arithmetic mean ± standard deviation (SD) of ten mice each group. Asterisks (*) indicate statistically significant differences (*P* < 0.05) in worm recovery of the immunized group compared to empty plasmid pcDNA3.1 and PBS control groups.

To evaluate the efficacy of the recombinant DNA vaccine strategy, the adult worm burden and the muscle larval burden were examined. After the challenge infection with *T. spiralis* muscle larvae, the mice immunized with pcDNA3.1-TsNd displayed a statistically significant 40.44% reduction in adult worm burden and a 53.9% reduction in muscle larvae (Figure 7), compared with PBS control group (F_adults_ = 43.265, F_larvae_ = 14.598, *P* < 0.05). The results demonstrated that the immunization with DNA vaccine induced the partial immune protection against challenge infection with *T. spiralis* larvae.

## Discussion

In some countries (such as Mexico, Argentine, Thailand, China, Vietnam and some Eastern European countries), pigs are frequently raised in backyards where pigs were often fed on raw swills [30,31], the prevalence of swine *Trichinlla* infection is higher in backyards and small farms of some countries. It is very difficult to change the farmers’ habits of pig breeding with raw swills. Thus, vaccines are urgently needed to prevent swine from *Trichinella* infection. In our previous study, the rTsNd was expressed in an *E. coli* expression system and the vaccination of mice with rTsNd induced a partial protective immunity against *T. spiralis* infection [13]. However, the expressed proteins in prokaryotic plasmids may differ from the native active protein in terms of structure and function [32].

DNA vaccination becomes more attractive because of its ability to induce a broad range of immune responses and long lasting immunity. However, DNA vaccines remain poorly immunogenic compared to protein vaccines [33]. An effective vaccine usually requires more than one immunization in the form of a prime-boost. In addition, the plasmid DNA is most commonly delivered by intramuscular inoculation or via the gene gun bombardment [7], but the later is practically inconvenient in terms of cost and simplicity. It was also reported that the intramuscular route was the best one among five delivery routes, including oral administration [34]. In the present study, to improve the efficacy of vaccination, the DNA encoding the protective antigen TsNd was cloned into the eukaryotic expression plasmid, and the recombinant TsNd DNA was used to immunize mice by intramuscular injection to induce the protective immunity against *T. spiralis* infection. The results showed that a TsNd DNA vaccine elicited a specific immune response and partial protection against *T. spiralis* challenge in mice. The mice immunized with pcDNA3.1-TsNd displayed a 40.44% reduction in intestinal adult worms and a 53.9% reduction in muscle larval burden. The parasite burden reduction observed in this study is similar with those from the previous reports [13,15,23].

The results of RT-PCR and IFT showed that the TsNd gene was transcribed and expressed in the BHK-21 cells, suggesting that the recombinant plasmid pcDNA3.1-TsNd was successfully constructed in this study. Antibody response analysis showed that intramuscular immunization with TsNd DNA induced not only the mixed systemic immune response but also the significant local mucosal IgA secretion in mice. Previous studies showed that secretory IgA (sIgA) played a crucial role in mucosal defense and may limit penetration of pathogens through the intestinal epithelium [35]. Protective immunity against *Trichinella* infection is obviously associated with the production of serological IgA [36]. IgA provides intestinal protection in a non-inflammatory manner. It has been demonstrated that non-specific and specific mucosal IgA responses, as a part of the overall immune response against the parasite, act against the infective larvae and adult worms of *T. spiralis* in the mouse intestine [37]. The rapid expulsion of adult worms from the intestines might be mediated by the sIgA against surface antigens of adult worms. Passive transfer of anti-*Trichinella* IgA monoclonal antibodies to naive mice conferred a high level of protection (more than 95%) against *Trichinella* infection [38]. The intestinal sIgA inhibited the *in vitro* fecundity of female worms [39]. Our results showed that vaccination with TsNd induced the production and secretion of TsNd-specific intestinal mucosal IgA. Normally, mucosal IgA production is strongly Th2-dependent; in particular, IL-10 is a major cytokine that enhance IgA responses [40]. In this study, the correlation of elevated intestinal mucosal IgA level with high level of cytokines IL-10 upon TsNd immunization indicates that the cytokines may promote intestinal mucosal IgA response.

The intramuscular vaccination with pcDNA3.1-TsNd induced specific antibody (IgG, IgA) responses, suggesting the good immunogenicity of TsNd. It has been shown that different delivery systems of vaccines influence the profile of immune responses. For example, oral immunization with Ts87 DNA vaccine [37] elicited a balanced Th1/Th2 antibody response, while subcutaneous immunization with recombinant *T. spiralis* proteins induced mainly Th2 antibody responses [41,42]. In this study, the cytokines IFN-γ, IL-2, IL-4, and IL-10 secretion by the splenocytes of mice immunized with TsNd DNA significantly increased after immunization. In addition, the IgG1 levels on week 4 and 6 after immunization were more obviously higher than that of IgG2a (*P* <0.01), but it was notable that IgG2a was also induced after immunization. The results suggested that the intramuscular vaccination of mice with TsNd DNA vaccine induced a Th1/Th2-mixed type of immune response. The Th1/Th2-mixed type of immune responses had been demonstrated to be important for protective immunity against *T. spiralis* infection [37,43].

Since *T. spiralis* is an antigenically complex parasite, the immune responses induced by single antigen vaccination may not be strong enough to combat the challenging infection. The vaccination of mice with TsNd induced a partial protective immunity against *T. spiralis* infection. Hence, the polyvalent vaccine including antigenic epitopes from *T. spiralis* different stages should be developed to improve further the efficacy of *T. spiralis* vaccine [29,44,45].

## Conclusions

The aim of this study was to observe the protective efficacy of intramuscular immunization with the TsNd DNA vaccine and its induced immune response. The results indicated that intramuscular immunization with TsNd DNA elicited a systemic Th1/Th2-mixed type of immune response and a strong mucosal IgA response, and produced a partial protection against *T. spiralis* infection in mice. However, the polyvalent vaccine including antigenic epitopes from *T. spiralis* different stages should be developed to improve further the efficacy of *T. spiralis* vaccine. Additionally, the exact mechanism of immune protection against *T. spiralis* infection needs to be investigated further.
